# The clinical phenotype of psychiatric-onset prodromal dementia with Lewy bodies: a scoping review

**DOI:** 10.1007/s00415-023-12000-w

**Published:** 2023-10-04

**Authors:** Chaminda Withanachchi Gunawardana, Elie Matar, Simon J. G. Lewis

**Affiliations:** Faculty of Medicine and Health, Brain and Mind Centre, School of Medical Sciences, University of Sydney, Level 2, M02G, 100 Mallett St, Camperdown, Sydney, NSW 2050 Australia

**Keywords:** Dementia with Lewy bodies (DLB), Prodromal, Psychiatric

## Abstract

**Background:**

Recent consensus research criteria have identified a ‘psychiatric onset’ form of prodromal dementia with Lewy bodies (DLB) characterised by prominent late-onset psychiatric symptoms. Although recognised as important to raise the index of diagnostic suspicion, evidence regarding this cohort was deemed too limited to impose formal criteria. We reviewed the published literature on psychiatric-onset DLB to identify key clinical characteristics and evidence gaps to progress our understanding of this entity.

**Methods:**

Medline, PubMed and Embase were searched for relevant articles containing longitudinal follow-up of patients initially presenting with a psychiatric illness who subsequently developed DLB according to the diagnostic criteria available at the time.

**Results:**

Two cohort studies (18 and 21 patients) along with 12 case series (13 cases) were identified totalling 52 patients (63% female). Initial psychiatric presentation occurred at a mean of 63 years (range 53–88), with depression being the most frequently reported psychiatric presentation (88%). Psychotic presentations were less common on presentation (11%) but became more prevalent throughout the prodromal period before the diagnosis of DLB (83%). Relapses of the psychiatric disease were common occurring in 94% (32/34) of patients. Parkinsonism, cognitive fluctuations, visual hallucinations, and REM sleep behaviour disorder were uncommonly reported at initial presentation (3.8%).

**Conclusions:**

Psychiatric-onset DLB is characterized by a female predominant relapsing–remitting psychiatric illness presenting with affective symptoms but later developing psychotic features prior to the onset of DLB. Additional prospective studies including other neurodegenerative cohorts with harmonised assessments are required to inform definitive diagnostic criteria for this condition.

## Introduction

When compared to Alzheimer’s dementia (AD), dementia with Lewy bodies (DLB) is associated with poorer outcomes, including increased mortality, caregiver burden and the need for earlier residential care [1]. These observations probably reflect the comorbid parkinsonian and neuropsychiatric features typical of DLB. The recently revised diagnostic criteria for DLB highlight an aggressive cognitive decline in combination with four core clinical features, namely parkinsonism, cognitive fluctuations, visual hallucinations and Rapid Eye Movement Sleep Behaviour Disorder (RBD) [2]. The presence of at least two of these core features or one core feature and an indicative biomarker (polysomnographic confirmation of REM sleep without atonia, abnormal iodine MIBG myocardial scintigraphy or reduced dopamine transporter uptake in the basal ganglia), allow for a diagnosis of probable DLB to be made clinically. In addition, the diagnostic criteria also recognise a range of supportive clinical features (e.g. constipation, hyposmia, and daytime somnolence) that can assist in making the diagnosis [2].

Given its mixed clinical phenotype, the accurate diagnosis of DLB can be delayed by several years [3]. This delay is likely to impact on successful disease-modifying strategies, which rely on a diagnosis at the earliest point in time [4]. Therefore, as seen with other neurodegenerative conditions, there has been an increasing emphasis on the identification of prodromal DLB. DLB is a synucleinopathy with a differential cell loss occurring across multiple neurotransmitters and neural pathways which can occur decades before clinical diagnosis [5]. Thus, prodromal DLB is likely to be characterised by a range of differing clinical presentations encompassing both cognitive and non-cognitive symptoms. To characterise these potential phenotypes, the International DLB Consortium proposed research criteria for prodromal DLB in 2020 [6]. They identified three subtypes of prodromal DLB characterised by (i) a mild cognitive impairment-onset; (ii) a delirium-onset; and (iii) a psychiatric-onset.

Whilst the evidence to support the research criteria proposed for prodromal mild cognitive impairment (MCI) with Lewy bodies (MCI-LB) was relatively robust, describing a non-amnestic phenotype often with associated RBD, the Consortium recognised that there was insufficient evidence to propose formal criteria for the delirium- and psychiatric-onset presentations.

Clearly, significant challenges exist when studying the symptomatic overlap between primary late-onset psychiatric disorders and the prodromal symptoms of DLB. For example, psychomotor slowing, antipsychotic-induced bradykinesia, and cognitive impairments are frequently seen in psychiatric conditions and may be very difficult to differentiate clinically from the early parkinsonism and neuropsychiatric impairment occurring in the context of an evolving DLB. Indeed, it has been reported that over 40% of patients who develop DLB present with a concurrent psychiatric symptom [7].

Despite the need to characterise the existence of a psychiatric-onset prodromal presentation of DLB, to the best of our knowledge, no previous reviews evaluating the current literature have been published. Thus, in this review, we aimed to identify those publications that have previously described patients who might satisfy the proposed psychiatric-onset DLB and determine the presence and frequency of clinical features and biomarkers that might allow us to better stratify the risk of their transition to dementia. It is hoped that establishing such a framework could help in proposing diagnostic guidelines and in the design of future prospective studies focussing on these patients.

## Methods

### Inclusion and exclusion criteria

Cohort or case studies that longitudinally followed up patients who presented after age 50 years with a primary psychiatric illness that subsequently developed DLB were included. Articles describing cross-sectional data without longitudinal follow-up or describing diagnosis of other Lewy body disorders (i.e. Parkinson’s disease dementia) were excluded.

### Search strategy

Terms for three psychiatric diseases (depression, psychosis and schizophrenia) were combined with terms encompassing dementia with Lewy bodies. Medline, Embase and PsycINFO were searched for English language for articles up to 11/2/2023. Keywords included (“depression”) OR Keyword (“Psychosis”) OR Keyword (“manic” OR “mania”) OR Keyword (“schizophrenia”) OR (“schizoaffective”) OR (“paranoid”) AND Keyword (“dementia with Lewy bodies”) OR Subject heading (“diffuse Lewy body disease”) AND Keyword (“dementia with Lewy Bodies”) OR Subject Heading (“dementia with Lewy Bodies”). Medline: Keyword (“depression”) OR MeSH Terms (“depression”) OR Keyword (“psychosis”) OR MeSH Terms (“psychotic disorders”) OR Keyword (“schizophrenia”) OR MeSH Terms (“schizophrenia spectrum” OR “other psychotic disorders” OR “schizophrenia” OR “paranoid schizophrenia” OR “schizophrenia” OR “catatonic schizophrenia” OR “Treatment-Resistant Schizophrenia, Disorganized”) AND Keyword (“dementia with Lewy Bodies”) OR (“Lewy Body Disease”). A parallel search strategy was also conducted, consulting reference lists and citing studies of included publications.

### Selection process

**Fig. 1:**
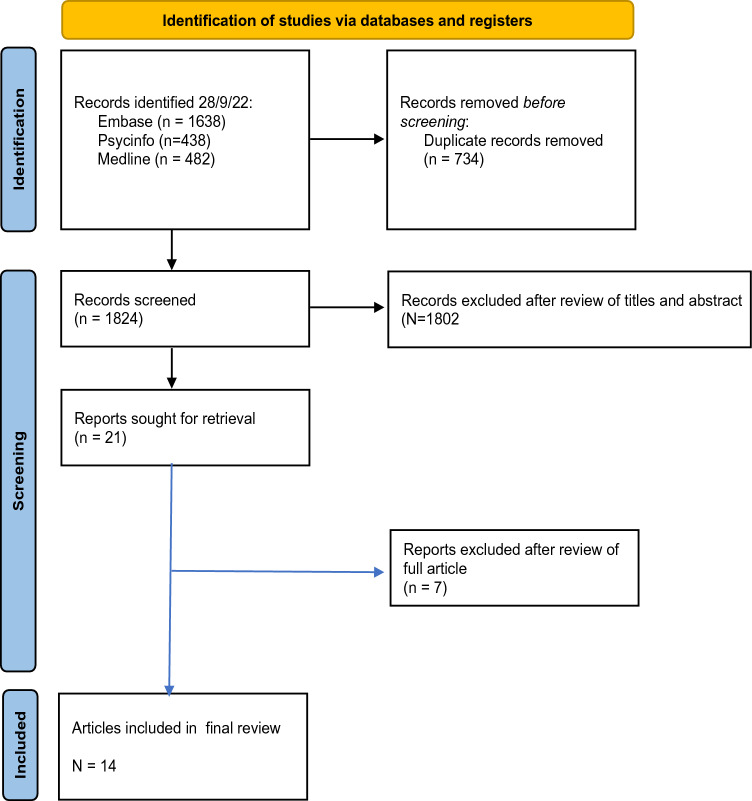
Flow Diagram of Database search and screening procedure

A total of 2558 results were retrieved, of which 734 duplicates were removed. The titles and abstracts of the remaining 1824 articles were reviewed (CWG). Of these, 21 articles were selected and reviewed in full. Seven articles were excluded as they described cross-sectional studies, patients who did not meet diagnostic criteria for dementia or patients with other Lewy body disorders (see Fig. 1).

### Data collection

Data were collated from the remaining 14 articles regarding psychiatric features, biomarkers, core, and supportive features utilising the existing guidelines in the Fourth DLB Consortium diagnostic criteria [8].

## Results

As shown in Table 1, of the 14 articles included in the final review, two were cohort studies and another 12 were case reports, which accounted for a total of 69 patients initially presenting with a psychiatric illness, of which 52 were subsequently diagnosed with DLB.

**Table 1:** Summary of patient demographics

Identifier	Type of study	Total number of patients	Age at onset of psychiatric diagnosis (years)	Gender	Psychiatric diagnosis	Time to conversion (Years)	Age at DLB onset (years)
Cohort 1 [9]	Cohort	35 (18 developed DLB)	Mean 61 ± 8.9 (Range 50–76)	Male 4 Female 14	Major depressive disorder	2–8	Not provided
Cohort 2 [11]	Cohort	21	Mean 6 ± 6.3 (Range 54–76)	Male 7 Female 14	Psychosis	Mean 9.1 ± 4.4	71 ± 6.1
CS1 [12]	Case report	1	88	Male	Major depressive disorder	1	89
CS2 [13]	Case report	1	75	Male	Major depressive disorder	< 1	76
CS3 [14]	Case report	1	53	Male	Schizoaffective disorder	6 years	59
CS4 [15]	Case report	1	78	Female	Major depressive disorder	3	81
CS5 [16]	Case report	1	88	Female	Oral cenesthopathy Delusional parasitosis/Depression	< 1	88
CS6 [17]	Case report	1	53	Male	Major depressive disorder	12 years	65
CS7 [18]	Case report	2	60 65	Male Male	Obsessive–compulsive disorder Obsessive–compulsive disorder	14 4	74 69
CS8 [19]	Case report	1	75	Female	Major depressive disorder	4	79
CS9 [20]	Case report	1	69	Male	Oral Cenesthopathy (Major depressive disorder)	< 1	70
CS10 [21]	Case report	1	56	Male	Major depressive disorder	12	68
CS11 [22]	Case report	1	78	Female	Panic attacks	5	83
CS12 [23]	Case report	1	62	Female	Major depressive disorder	14	76
Total		52	Mean 63	Male 19		Mean 8 ± 5 (34 patients excluding Cohort 1)	Mean 73 ± 7.5 (34 patients Excluding Cohort 1)
			Range 53–88	Female 33			

The largest study identified in this review was conducted by Takahashi et al. [9], which recruited 35 patients with major depressive disorder (MDD) diagnosed according to the Diagnostic and Statistical Manual of Mental Disorders (DSM)-IV-TR) [9] (mean age 71 ± 9.7, males 8, females 27). The primary purpose of this study was to evaluate the utility of the ventilatory response to hypercapnia (VRH) as a potential novel biomarker of autonomic dysfunction in patients that might have an early neurodegenerative condition. The study included patients (over 50 years) who required admission into a psychiatric unit with a Mini-Mental State Examination score of over 24/30 and the presence of bradykinesia but the absence of other extrapyramidal signs (i.e. scores of ≥ 1 for bradykinesia but 0 for the remaining 13 items on the Unified Parkinson’s Disease Rating Scale part III [10]). The initial presentation of MDD occurred in these patients prior to their recruitment into this study. The interval between initial presentation with MDD and recruitment into the study was not specified. Patients underwent a detailed neuropsychological assessment battery, as well as autonomic function testing that included ^123^I-meta-iodobenzylguanidine (MIBG) scintigraphy, heart rate variability, orthostatic hypotension assessments and VRH. These patients were followed up annually and assessed for mood and cognitive state. They required at least three annual assessments to be included in the study. The examination period for this cohort ranged between 3 and 8 years during which 18/35 patients were diagnosed with DLB (mean age: 61 years; range 50–76 years; male: 4, female: 14).

The second cohort study evaluated here was conducted by Utsumi et al*.* [11]. This study described both the clinical and biomarker characteristics of 21 patients (mean age 62 ± 6.3 years, range 54–76 years, 7 males, 14 females) who presented with a psychotic illness requiring at least one hospital admission who went on to develop DLB. Patients needed to demonstrate no overt impairment in their activities of daily living or functional status for at least 2 years after their first psychotic episode. To be recruited into this cohort, patients needed to have at least one positive radiological biomarker that was indicative of DLB (i.e. (123I-N-ω-fluoropropyl-2β -carbomethoxy-3β-(4-iodophenyl) nortropane single photon emission computed tomography (FP-SPECT) and/or ^123^I-meta-iodobenzylguanidine (MIBG) scintigraphy) or a brain perfusion SPECT, as a supportive biomarker of DLB. This study presented data on psychiatric symptoms, core features of DLB and biomarker results. These 21 patients developed DLB after a mean prodromal period of 9.1 (± 4.5, range 2–19) years at a mean age of 71 years (± 6.1, range 60–82).

The remaining 12 articles were case studies that described a total of 13 patients (5 females, 8 males). Their mean age at presentation of their first psychiatric illness was 69 years (± 12; range 53–88). This group had a mean prodromal period of 6.2 years (± 5.5, range 1–17) and were diagnosed with DLB at a mean age of 75 years (± 9.0, range 59–89).

Altogether, these 14 articles described 52 patients who developed DLB (mean age at initial psychiatric presentation 63 years, range 53–88, males 19: females 33). Takahashi et al. [9] did not report the interval between first psychiatric presentation and the diagnosis of DLB. However, for the remaining 34 patients, the mean time for conversion to DLB was 8 (± 5) years from the onset of first psychiatric symptoms to a diagnosis of DLB, which occurred at a mean age of 73 (± 7.5) years. Both patients who presented with the core clinical feature (CS1 and CS2, see below), transitioned to DLB within 1 year. The available data showed no significant difference in age at presentation or time to conversion from initial onset of psychiatric illness to the development of DLB between the males and females.

The study by *Utsumi *et al. [11] was the only manuscript that reported a comparator group of patients who presented with MDD but did not transition to DLB over the course of the study. In this study, 18 patients who converted to DLB, compared to the 17 who did not, were younger at the onset of their psychiatric illness, although this difference was not statistically significant (61 ± 8.9 years *versus* 69 ± 9.2 years). A female predominance was seen in both groups (13/17 for DLB non-converters *versus* 14/18 for converters).

### Psychiatric symptoms

For the purpose of this review, psychotic features were subdivided into hallucinations, delusions or catatonia. Similarly, affective features were subdivided into depression and anxiety.

#### Individual studies

The patients included in the study by Takahashi et al., (Cohort 1 in Table 2) were recruited on or after admission to a psychiatric unit. The initial presentation with a psychiatric illness could have occurred up to 2 years prior to this admission (and therefore recruitment into this study). All patients had an initial presentation with major depressive disorder (MDD) and the article did not detail when subsequent psychotic symptoms occurred. Therefore, the temporal progression of psychiatric symptoms could not be accurately assessed in this study.

**Table 2:** Psychiatric features of prodromal DLB

	Initial presentation only					Prodromal phase					
	Hallucinations	Delusions	Catatonia	Depression	Anxiety	Hallucinations	Delusions	Catatonia	Depression	Anxiety	Multiple episodes (Total patients)
Cohort 1 [9]	Psychotic features 0 (18)			18 (18)	Not reported	Psychotic features 13 (18)			18 (18)	Not reported	Not reported
Cohort 2 [11]	2 (21) Auditory	0 (21)	0 (21)	18 (21)	Not Reported	Psychotic features 21 (21)				Not reported	20 (21)
						Total 16 (21) Visual only 4 (21) Auditory only 4 (21) Both 8 (21)	12 (21)	9 (21)	21 (21)	Not reported	
CS1 [12]	Yes—Visual	Yes -Poverty	No	Yes	No	Yes—Visual	Yes	Yes	Yes	No	No
CS2 [13]	Yes—Visual	Yes - Persecutory	No	Yes	Yes	Yes- Visual	Yes	No	Yes	Yes	Yes
CS3 [14]	Yes- Auditory	Yes -Persecutory	No	No	No	Yes- Auditory	Yes	No	Yes	No	Yes
CS4 [15]	No	No	No	Yes	Yes	No	No	No	Yes	Yes	Yes
CS5 [16]	No	No	No	Yes	No	No	Yes	No	Yes	No	Yes
CS6 [17]	No	No	No	Yes	No	Yes Visual	No	No	Yes	No	Yes
CS7 [18] 1 2	No No	No Yes—Somatic	No No	No Yes	Yes Yes	No Yes- Visual	No Yes	No No	Yes Yes	Yes Yes	Yes Yes
CS8 [19]	No	No	No	Yes	Yes	Yes- Visual	No	No	Yes	Yes	Yes
CS9 [20]	No	No	No	Yes	No	No	No	No	Yes	No	Yes
CS10 [21]	No	No	No	Yes	No	Yes – Visual	No	No	Yes	No	Yes
CS11 [22]	No	No	No	No	Yes	No	No	No	Yes	Yes	Yes
CS12 [23]	No	No	No	Yes	No	Yes—Visual	No	No	Yes	Yes	Yes
	Psychotic features 6 (52)–11%					Psychotic features 43 (52) 83%					
Total	5 (34) 15% Auditory 3 (34) 8.8% Visual 2 (34) 5.9%	4 (34) 12%	0 (34)–0%	46 (52)–88%	6 (13)–46%	Visual 11 (34)–32% Auditory 5 (34)–15% Both 8 (34)–23%	17 (34) 50%	10 (34) 29%	52 (52) 100%	7 (13) 54%	32 (34) 94%

() Total number of patients with symptoms.

This study subdivided patients based on whether they displayed “psychotic” and/or “melancholic” features but did not provide further delineation of these features or when they specifically occurred over the course of the illness. However, psychotic symptoms were described in 72% (13/18) of those patients who were ultimately diagnosed with DLB compared to just 41% (7/17) of those who did not transition.

The study by Utsumi et al. (Cohort 2 in Table 2) reported 21 patients who developed DLB after initially presenting with a psychiatric illness that resulted in at least one hospitalisation for psychosis. The temporal onset of both affective and psychiatric symptoms was described. Of these 21 patients, 86% (18/21) initially presented with depression, 9.5% (2/21) with auditory hallucinations and 4.8% (1/21) with mania. Significantly, 93% (13/14) of females within this cohort reported hallucinations occurring in any sensory domain compared with just 43% of male patients (3/7). Similarly, delusions were more prevalent in females (10/14–71%) compared to males (2/7–28%).

The 13 patients described in the 12 case studies had a greater variety of primary psychiatric diagnoses including major depressive disorder (8 patients), schizoaffective disorder (1 patient), obsessive compulsive disorder (2 patients), delusional parasitosis (1 patient) and panic disorder (1 patient).

#### Group analysis

Across all the reported cases that transitioned to DLB, depression was the most common symptom both at initial presentation (46/52; 88%) and during the prodromal phase up to the time of a DLB diagnosis (52/52; 100%). Anxiety was only reported in the case studies and occurred in 7 of 13 patients.

The studies included in this review used different terminology to report psychotic features. Therefore, it was difficult to develop a cohesive model for these symptoms. However, it was clear that florid psychotic features were rare at initial presentation occurring in only 11% of patients (6/52). Of these six cases, two suffered from isolated auditory hallucinations (both were female and from Cohort 2), three suffered from both hallucinations and delusions whereas one suffered from isolated delusions (4 patients from case studies CS1, CS2, CS3, and CS7, all of whom were male).

Whilst the study by Takahashi et al., reported on psychotic symptoms in 13 patients who converted to DLB, it is not clear when these symptoms evolved during the prodromal period. Furthermore, this study did not define individual psychotic symptoms (e.g. hallucinations, delusions and catatonia), which were thus not included in the detailed analysis of psychotic features presented here.

Psychotic symptoms became more prevalent during the course of the prodromal period and were reported in 83% of patients (43/52) prior to their diagnosis of DLB. Of these psychotic symptoms, hallucinations were the most common, occurring in 71% of patients where data were available (24/34). Isolated visual hallucinations were the commonest manifestation occurring in 32% of patients (11/34), followed by visual and auditory (8/34; 23%) and isolated auditory phenomena (5/34; 15%). Delusions occurred in 50% of patients where the data was available (17/34), whilst catatonia was reported in 29% of cases (10/34).

Data on the recurrence of psychiatric symptoms were available for 34 patients (21 patients in Cohort 2 and 13 from the 12 case studies). Significantly, 94% of these patients (32/34) had two or more recurrences of their psychiatric illness. This included 95% (20/21) from Cohort 2 and 92% (12/13) of patients from the case studies.

It is clear from both Cohort 2 and the case studies that the psychiatric diagnosis at each recurrence changed in nature. Aside from the above-described psychiatric features (depression, hallucination, delusions, and catatonia) four patients also demonstrated delirium-like states (7.7%). Of note all 52 patients that ultimately transitioned to DLB required at least one hospitalisation for the management of their psychiatric illness.

### Core and supportive features

The data on core and supportive clinical features is presented in Table 3.

**Table 3:** Core and Supportive features

	First presentation					Prodromal phase				
Identifier	Core				Supportive features	Core				Supportive features
	Parkinsonism	Fluctuations	Recurrent VH	RBD		Parkinsonism Symptoms	Fluctuations	Recurrent VH	RBD	
Cohort 1 [9]	0 (18)	Not reported	Not reported	Not reported	Not reported	18 (18) Bradykinesia 18	Not reported	Not reported	Not reported	Orthostatic hypotension – 9 (18) Neuroleptic sensitivity 13 (18)
Cohort 2 [11]	0 (21)	0 (21)	0 (21)	0 (21)	Not reported	6 (21) 8 (21)^a^	0 (21) 13 (21)^a^	9 (21) 3 (21)^a^	5 (21) 0 (21)^a^	Not reported
CS1 [12]	No	No	Yes	No	No	Yes^a^	Yes	Yes	Yes	Neuroleptic sensitivity
CS2 [13]	No	No	Yes	Yes	Urinary Incontinence	Yes Rigidity Bradykinesia	Yes^a^	Yes	Yes	Urinary Incontinence
CS3 [14]	No	No	No	No	No	Yes Not specified	Yes^a^	Yes^a^	No	No
CS4 [15]	No	No	No	No	No	Yes Rigidity Bradykinesia	Yes^a^	No	Yes	Sense of presence
CS5 [16]	No	No	No	No	No	Yes Tremor Rigidity Bradykinesia	No	No	No	No
CS6 [17]	No	No	No	No	No	Yes^a^ Tremor Bradykinesia	No	Yes^a^	No	No
CS7 [18] 1 2	No No	No No	No No	No No	No No	Yes Bradykinesia No	Yes^a^ No	No Yes	Yes Yes	Falls, Postural instability Neuroleptic sensitivity Constipation Neuroleptic sensitivity
CS8 [19]	No	No	No	No	No	Yes^a^ (Not specified)	No	No	Yes	None
CS9 [20]	No	No	No	No	No	No	No	No	No	None
CS10 [21]	No	No	No	No	No	Yes^a^ Bradykinesia Tremor	Yes^a^	Yes^a^	No	
CS11 [22]	No	No	No	No	No	Yes Bradykinesia Tremor	Yes	No	No	Syncope Constipation
CS12 [23]	No	No	No	No	Anosmia Constipation Sweating during sleep	Yes (Not specified)	No	Yes^a^	Yes	Anosmia Constipation
Total	0 (52)	0 (34)	2 (34) 5.9%	1 (34) 2.9%		31 (52) 12 (52)^a^ Total 43 (52) 83%	2 (34) 18 (34)^a^ Total 20 (34) 59%	12 (34) 7 (34)^a^ Total 19 (34) 56%	11 (34) 0 (34)^a^ Total 11 (34) 32%	

^a^Occurred at or after DLB diagnosis

The study by Takahashi et al. (Cohort 1) [9] required patients to have isolated bradykinesia (parkinsonism) for enrolment into the study and it was not clear when bradykinesia first occurred. Given the uncertainty, it was assumed that bradykinesia was not present at the time of their initial psychiatric diagnosis. Similarly, whilst the study by Utsumi et al. (Cohort 2) [11] reported on all core features, the paper did not specify which of these symptoms (if any) were present at the time of initial psychiatric diagnosis.

Having made these assumptions, core symptoms were present during the initial psychiatric presentation in only 4% of the patients (2/52) as reported in CS1 and CS2 (Table 3). Parkinsonism was the commonest reported core symptom in the prodromal phase occurring in 83% of patients (43/52) with bradykinesia being the most frequent motor symptom, seen in 56% (24/43). Only Cohort 2 and the case studies (total 34 patients) reported on the remaining core features. Of these, cognitive fluctuations occurred in 59% of patients (20/34), recurrent visual hallucinations in 56% (19/34) and RBD in 32% (11/34). Where data was available, cognitive fluctuations occurred only at the time of DLB diagnosis in 90% of patients (18/20), whereas parkinsonism, recurrent visual hallucinations and RBD, tended to occur earlier throughout the prodromal period.

Supportive features were variably reported in these articles. The study by Takahashi et al*.,* (Cohort 1) reported on orthostatic hypotension and neuroleptic sensitivity, both of which occurred more frequently in the patients who converted to DLB [orthostatic symptoms 9/18–50% versus 3/17–18%; neuroleptic sensitivity 13/18–73% versus 2/17 (12%)]. The study by Utsumi et al. (Cohort 2) reported on symptoms suggestive of neuroleptic sensitivity in 4 patients administered with tranquillisers. Similarly, neuroleptic sensitivity was reported in 3 of the case studies.

### Biomarkers

As shown in Table 4, MIBG was the most commonly performed biomarker, followed by cerebral perfusion imaging and dopamine transporter imaging. In total, MIBG scans were abnormal in 54% of patients (14/26) during the prodrome and had a higher sensitivity on or after the clinical diagnosis of DLB was made (8/11–73%). 123I-N-fluoropropyl-2β-carboxymethoxy-3β-(4-iodophenyl) nortropane SPECT (FP-CIT) was utilised in 16 patients in the prodromal stage where it was abnormal in 81% (13/16). A further 11 patients who underwent FP-CIT at the time of DLB diagnosis returned an abnormal scan. Perfusion imaging consisted of Technetium-99m hexamethyl propylenamine oxime (HMPAO) SPECT (CT-SPECT) and fluorodeoxyglucose (FDG)-positron emission tomography (FDG-PET). CT-SPECT was positive in 80% of patients (12/15) during the prodromal phase and had similar sensitivity at the time of DLB diagnosis (8/10–80%).

**Table 4:** Biomarkers

Identifier	Prodromal phase				At diagnosis			
	Dopamine transporter imaging positive(Total)	Perfusion imaging positive(Total)	MIBG positive(Total)	Other	Dopamine transporter imaging positive(Total)	Perfusion imaging positive(Total)	MIBG positive (Total)	Other
Cohort 1 [9]	-	-	11 (18)	VRH–18 (18)				
Cohort 2 [11]	FP-CIT 12 (13)	CT-Spect 11 (13)	3 (7)	VRH 18/18	FP-CIT 5 (5)	CT-Spect 4 (6)	2 (5)	
CS1 [12]	NA	NA	NA	NA	NA	NA	NA	EEG – Normal
CS2 [13]	NA	NA	NA	NA	NA	NA	+	NA
CS3 [14]	NA	CT Spect -ve	NA	NA	NA	CT Spect + ve	+	MRI—Normal
CS4 [15]	FP-CIT + ve	NA	NA	NA	NA	NA	NA	NA
CS5 [16]	NA	NA	NA	NA	NA	CT Spect + ve	+	MRI—Non specific changes
CS6 [17]	FP-CIT -ve	FDG-PET + ve (Cingulate island sign)	NA	EEG – Normal	FP-CIT + ve	NA	NA	NA
CS7 [18] 1 2	NA NA	NA NA	NA NA	NA NA	FP-CIT + ve FP-CIT + ve	FDG-PET + ve CT Spect + ve	NA NA	MRI—Preservation of medial temporal lobe MRI—Non specific change
CS8 [19]	NA	NA	NA	NA	FP-CIT + ve	NA	+	PSG showing REM sleep without atonia
CS9 [20]	NA	NA	NA	NA	NA	FDG-PET + ve	NA	NA
CS10 [21]	NA	NA	NA	NA	FP-CIT + ve	CT Spect + ve	+	NA
CS11 [22]					Dopa PET + ve			MRI—sparing of hippocampi EEG-Compressed spectral arrays—variability of dominant frequency -2–3 Hz
CS12 [23]	FP-CIT -ve	CT Spect + ve	-ve	PSG showing REM sleep without atonia	FP-CIT + ve	NA	+	
Positive rate	FP-CIT 13 (16) 81%	CT Spect 12 (15) 80% FDG- PET 1 (1) 100%	14 (26) 54%	–	FP-CIT 11 (11) 100% Dopa Pet 1 (1) 100%	CT Spect 8 (10) 80% FDG-PET 2 (2) 100%	8 (11) 73%	

*FP-CIT* 123I-N-fluoropropyl-2β-carboxymethoxy-3β- (4-iodophenyl) nortropane SPECT, *Dopa-PET* 18F-dihydroxyphenylalanine (DOPA) Positron Emitted Tomography (PET), *CT* Spect: Technetium-99m hexamethyl propylenamine oxime (HMPAO) SPECT, *FDG-PET* Fluorodeoxyglucose (FDG)-positron emission tomography (FDG-PET), *MIBG* ^123^I-meta-iodobenzylguanidine scintigraphy, *EEG* Electroencephalogram, PSG: Polysomnography

Only two patients (CS8 and CS12) had polysomnography (PSG), which both demonstrated REM sleep without atonia, although only one of these cases described dream enactment suggestive of RBD. All patients included in the study by Utsumi et al*.,* (Cohort 2) underwent magnetic resonance or computed tomography imaging, although the results of these were not described in the article. Additionally, five patients from the case studies underwent MRI scans and two of these were reported as showing relative sparing of medial temporal lobes, a supportive biomarker in the diagnosis of DLB [2]. None of these imaging studies mentioned insular thinning, which has also been proposed as a potential biomarker in prodromal DLB [6].

## Discussion

This review reports the existing literature in relation to the possible existence of a psychiatric-onset presentation of prodromal DLB. To date, a total of 14 articles have reported 52 cases of non-demented patients presenting with an initial psychiatric diagnosis (> 50 years) who developed DLB over a variable period of 1–19 years (mean 8 years). Overall, most of these patients had recurring bouts of psychiatric symptoms that typically presented with depression, but commonly developed psychotic features before manifesting severe cognitive decline and the core features that led to the eventual diagnosis of DLB.

Strikingly, whilst there is a well-known male predominance in the prevalence of DLB, these studies highlight that patients presenting with prodromal psychiatric features were disproportionately female (63%). This female predominance is typically seen in late-onset depression and psychosis [24, 25] but there are few longitudinal studies that have tracked the progression of potential neurodegeneration in these cases. Indeed, it has recently been proposed that female DLB patients are more likely to present with psychotic symptoms compared to males who typically present more frequently with RBD [26].The majority of patients included in this review were Japanese (Cohort 1 and 2 totalling 39 patients, 75%) which accounted for the female predominance. Previous studies of Japanese DLB patients have noted a female predominance [26, 27], and therefore it is unclear whether ethnicity accounts for the unexpected gender difference. The data presented here also point towards psychiatric-onset DLB being a female-predominant phenotype compared to that of the MCI-LB prodrome, which has been shown to be more common in males [28].

This study highlights the difficulty in identifying patients with prodromal DLB who initially present with a psychiatric illness based purely on clinical clues. Core features tended to occur later in the course of the disease, and the majority of patients in the case studies initially were managed in the community. It is well known that mimics of the core symptoms reported in DLB can occur in patients with psychiatric disorders. For example, the presence of psychomotor retardation or paratonia can be mistaken for parkinsonism. Similarly, commonly used antidepressants such as selective serotonin reupdate inhibitors can precipitate RBD [29].

The utility of biomarkers in identifying these patients requires further evaluation. Whilst FP-CIT and MIBG were the most common biomarkers utilised in this study with sensitivities close to those demonstrated in previous studies [30], we are not aware of any work that has investigated the utility of these biomarkers in isolated depression or psychosis, where there has been a lower index of suspicion. Furthermore, studies to date have focussed on differentiating AD from DLB, rather than identifying patients who may progress to DLB from an isolated psychiatric disease.

This review has a number of limitations which impacts the confidence we can have in our conclusions. Firstly, the two cohort studies accounted for 75% (39/52) of patients and selection bias resulting from their respective inclusion criteria, may have skewed the presence or absence of clinical features (i.e. presence of bradykinesia in Cohort 1 and psychotic symptoms in Cohort 2). Furthermore there was inconsistent reporting relating to the timing and number of core features and biomarkers between studies. Interpretation of psychiatric features was also challenging, given the differences in terminology and reporting that has occurred over time with the evolution of psychiatric diagnostic criteria. Similarly, the lack of a standardised assessment battery and the reliance on clinical criteria may also have impacted on the ability to evaluate cases accurately. Together, these limitations emphasise the need for more high-quality and ideally multicentre prospective studies with larger case numbers undergoing standardised assessments. Unfortunately, given the lack of a comparison arm in most of the published studies, it is not possible to comment on the predictive ability of individual affective symptoms (depression, anxiety, apathy) in relation to future DLB risk. Future studies looking at patient groups with well-characterised affective symptoms measured by validated scales, but not necessarily carrying a formal diagnosis of anxiety or depression may also offer further insights into the psychiatric prodrome of DLB.

There have been no studies to date investigating the neuropathology of Prodromal DLB. Studies investigating the neuropathology of late-life depression have shown a mixture of Alzheimer's (Amyloid plaques), Lewy bodies and vascular pathology [31–33], although none of these studies describe patients who had developed DLB. Work characterising the neuropathology of patients with psychiatric-onset DLB to determine the potential influence of mixed comorbidities (e.g. synucleinopathy, amyloid, tau) could provide a greater understanding of the underpinning neurobiological processes.

Despite the limitations, the results above provide some practical starting points that may be useful for clinicians to increase their awareness of this condition and assist recruitment and design of future studies. Firstly, the onset of new psychiatric symptoms in patients (especially females), over the age of 50 years should trigger the routine investigation for other core and supportive clinical features that might otherwise not be considered, such as parkinsonism, RBD, hyposmia, orthostatic hypotension, and constipation [34, 35]. Secondly, relevance should be given to such patients who experience multiple or recurrent bouts of psychiatric symptoms and/or delirium-like episodes. Thirdly, the transition from presenting with affective to psychotic features appears to be of particular importance in the risk of developing DLB and should raise the suspicion in treating clinicians for underlying neurodegeneration. In addition, the presence of any of the above features should trigger caution in physicians with respect to the prescribing of typical antipsychotics that may increase morbidity. Finally, we are entering a new era where we may be able to consider more sensitive biomarkers of disease from techniques such as α-synuclein seed amplification [36, 37], which may become more routine than established tests such as PSG, MIBG or FP-CIT(DaTscan).

In summary, much work is still needed to improve our understanding of this entity given the relative paucity small number of cases reported in the literature to date. Advancing this field will take a concerted international effort, and interdisciplinary collaboration between psychiatry, primary care and neurology will also be essential. It is hoped that a greater understanding of prodromal DLB will lead to earlier and more accurate diagnosis with the potential for more meaningful treatment strategies, as well as avoidance of morbidity associated with inappropriate neuroleptic prescription in this cohort.
